# The Structure and Role of Intramuscular Connective Tissue in Muscle Function

**DOI:** 10.3389/fphys.2020.00495

**Published:** 2020-05-19

**Authors:** Peter P. Purslow

**Affiliations:** Centro de Investigacion Veterinaria de Tandil, Facultad de Ciencias Veterinarias, Universidad Nacional del Centro de la Provincia de Buenos Aires, Tandil, Argentina

**Keywords:** intramuscular connective tissue, extracellular matrix, collagen, endomysium, perimysium, muscle, mechanotransduction

## Abstract

Extracellular matrix (ECM) structures within skeletal muscle play an important, but under-appreciated, role in muscle development, function and adaptation. Each individual muscle is surrounded by epimysial connective tissue and within the muscle there are two distinct extracellular matrix (ECM) structures, the perimysium and endomysium. Together, these three ECM structures make up the intramuscular connective tissue (IMCT). There are large variations in the amount and composition of IMCT between functionally different muscles. Although IMCT acts as a scaffold for muscle fiber development and growth and acts as a carrier for blood vessels and nerves to the muscle cells, the variability in IMCT between different muscles points to a role in the variations in active and passive mechanical properties of muscles. Some traditional measures of the contribution of endomysial IMCT to passive muscle elasticity relied upon tensile measurements on single fiber preparations. These types of measurements may now be thought to be missing the important point that endomysial IMCT networks within a muscle fascicle coordinate forces and displacements between adjacent muscle cells by shear and that active contractile forces can be transmitted by this route (myofascial force transmission). The amount and geometry of the perimysial ECM network separating muscle fascicles varies more between different muscle than does the amount of endomysium. While there is some evidence for myofascial force transmission between fascicles via the perimysium, the variations in this ECM network appears to be linked to the amount of shear displacements between fascicles that must necessarily occur when the whole muscle contracts and changes shape. Fast growth of muscle by fiber hypertrophy is not always associated with a high turnover of ECM components, but slower rates of growth and muscle wasting may be associated with IMCT remodeling. A hypothesis arising from this observation is that the level of cell signaling via shear between integrin and dystroglycan linkages on the surface of the muscle cells and the overlying endomysium may be the controlling factor for IMCT turnover, although this idea is yet to be tested.

## Introduction

Intramuscular connective tissue plays a critical role in the development and growth of muscle tissue and its quantity and distribution vary greatly between muscles with different functional properties. Yet surprisingly, relatively little is known about the properties and adaptation of IMCT in comparison with the knowledge of muscle function and plasticity (Kjaer, 2004). There have been several different general terms and abbreviations used to describe the extracellular matrix (ECM) within muscle. The term “intramuscular connective tissue” (IMCT) will be used consistently here.

This article reviews the structure and roles of connective tissue structures within skeletal muscle tissues, with an emphasis on recent developments and remaining questions. This subject has a rich history; connective tissue structures surrounding individual muscle fibers muscle were first described by Bowman (1840). There have been substantial investigations of the variations in amount and spatial distribution of IMCT between different muscles of farm animals because of the large influence IMCT has on the sensory qualities of muscle when cooked and eaten as a food (Lehmann, 1907; Mitchell et al., 1927; Ramsbottom et al., 1945; Strandine et al., 1949; Loyd and Hiner, 1959; Rowe, 1981). The role of covalent crosslinks between collagen molecules in modifying the properties of IMCT with increasing physiological age has also been well-documented. From the observation that newly synthesized collagen was the most easily extracted, Jackson and Bentley (1960) rationalized that the degree of collagen stabilization by covalent crosslinking increased with time post synthesis. Newly synthesized fibrillar collagen is stabilized divalent crosslinks between lysine and hydroxylysine residues in the non-helical portion of the molecule and hydroxylysine residues in the helical portion of adjacent molecules (Shimokomaki et al., 1972). A slow process of condensation between these divalent crosslinks to produce more stable trivalent crosslinks (Eyre, 1987) is associated with stronger and stiffer IMCT. Gao et al. (2008) demonstrated that epimysium from the tibialis anterior muscle of rats became stiffer with animal age. A complete description of lysyl-derived crosslinking of collagen is given by Scott et al. (2012) and will not be discussed further here, but is relevant to considerations of the influence of IMCT on changes in muscle functionality in human aging (Kragstrup et al., 2011).

## The Structural Components of Intramuscular Connective Tissue

There are numerous comprehensive reviews of the structure of IMCT in the literature. Purslow (2014) tabulated 17 previous reviews of IMCT structure, mechanical properties, development, turnover, and function. Gillies and Lieber (2011) also review some pathological changes in IMCT in addition to its basic structure, composition and properties. With such a wide choice of excellent sources it is unprofitable to repeat an in-depth review of basic IMCT structure here and, instead, a summary of IMCT structure will be provided together with a discussion of what is relatively new information, and questions that remain to be clarified.

**FIGURE 1:**
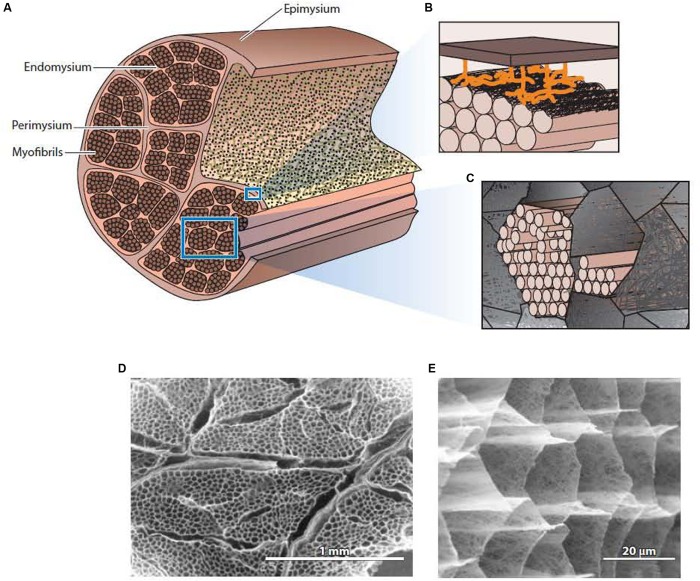
The general structure of intramuscular connective tissue. **(A)** Schematic diagram showing the general arrangement of the epimysium, perimysium, and endomysium within muscle. **(B)** Schematic diagram depicting the sparse junction zones between the thick perimysium and the endomysium of muscle fibers in the surface layer of the fascicle. **(C)** Schematic diagram showing myofibrils of an individual muscle cell residing in the honeycomb network of the endomysium. **(D)** Low magnification scanning electron micrograph of IMCT structures in muscle after treatment with NaOH to remove myofibrillar proteins and proteoglycans. The thicker perimysium is seen surrounding the honeycombed endomysial network within a fascicle. **(E)** A higher magnification view of the endomysial network after NaOH treatment. From Purslow (2014), with permission. Panels **(D,E)** from Purslow and Trotter (1994), with permission.

The general structure of IMCT is summarized in Figure 1. Each muscle is an individual organ that is surrounded by an outer ECM layer, the epimysium (Schmalbruch, 1985). Internally, the muscle is divided onto fascicles or bundle of muscle fibers by a continuous network of connective tissue structures termed perimysium. The perimysial network is connected to the epimysium at the surface of the muscle. Within each fascicle or fiber bundle, another continuous network structure, the endomysium, lies between individual muscle fibers. As emphasized elsewhere (Purslow and Delage, 2012), it is common for previous literature to describe the endomysium and perimysium as tubes or sheaths that surround each fiber and fascicle, respectively, giving the impression that these “sheaths” individually surround and separate each fiber and fascicle. In reality, (as is evident from Figure 1E) the endomysium forms a continuous three-dimensional network throughout the fascicle, provided a connection between adjacent muscle fibers rather than separating them. The perimysium is also a continuous three-dimensional network that runs the length and breadth of the muscle, linking the muscle fascicles that lie in the interstices of this network.

It is a common assumption that muscle fibers typically run the entire length of a muscle fascicle, inserting onto tendons by myotendinous junctions at both ends. However, numerous studies on a wide variety of species have shown that many muscles have muscle fibers that do not span then entire fascicle, Muscles with non-spanning or intrafascicularly terminating muscle fibers are actually quite common (Gaunt and Gans, 1993; Trotter, 1993; Hijikata and Ishikawa, 1997). Hijikata and Ishikawa (1997) distinguish between non-spanning fibers that terminate on tendinous insertions in some muscles (e.g., mammalian rectus abdominis) and those having short muscle fibers that taper down at each end and terminate within the fascicle, with no connection to the tendons or tendinous insertions (intrafascicularly terminating fibers), the series-fibred muscle. For example, the main locomotory muscles of birds (pectoralis muscles) are series fibers in 63 species studied, from hummingbirds to turkeys (Gaunt and Gans, 1993). In series fibred muscles, connections between fibers via the endomysium are the only possible route for contractile force transmission.

### Structure of the Endomysium

Each muscle fiber (cell) is bounded by its plasmalemma (sarcolemma) and, external to this, a 50 nm thick basement membrane layer comprized of non-fibrous type IV collagen and laminin in a proteoglycan matrix. Lying between the two basement membranes of two adjacent muscle fibers, the fibrous network layer of the endomysium forms a continuum between the two basement membranes. Schmalbruch (1974) estimated that this network layer can be between 0.2 and 1.0 μm in thickness. The fine collagen fibers that make up the bulk of the network layer, together with an amorphous proteoglycan matrix, comprise a planar feltwork of quasi-randomly orientated, wavy fibers (Figure 1E). Transmission electron micrographs of cross sections through the endomysium show that all the collagen fibers run in the plane parallel to the muscle fiber surfaces (Trotter and Purslow, 1992). The preferred orientation of the collagen fibers in the endomysial network changes with muscle sarcomere length, but at all sarcomere lengths the great majority of collagen fibers are still wavy (Purslow and Trotter, 1994) and therefore relatively compliant in tension.

### Structure of the Perimysium

**FIGURE 2:**
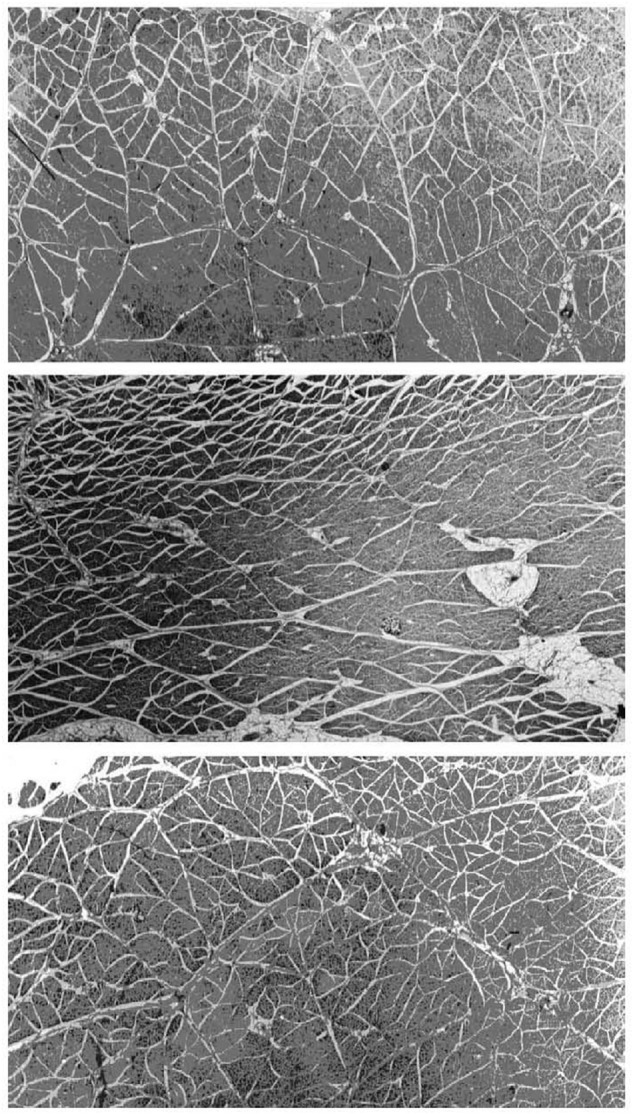
Micrographs of large transverse sections of three muscles from the same (bovine) animal, showing differences in the division of muscles into fascicles by perimysium. Top panel: pectoralis profundus; middle panel: sternocephalicus; bottom panel: rhomboideus cervicus. Differences in fascicle shape, size, and perimysial thickness can be seen between muscles and within each muscle. White gaps visible between fascicles are shrinkage artifacts (separating perimysium from the endomysium of surface muscle fibers) produced by fixation. Adapted from Purslow (2005).

The perimysium is described as a well-ordered criss-cross lattice of two sets of wavy or crimped collagen fiber bundles in a proteoglycan matrix, with each of the two parallel sets of wavy fibers at angle symmetrically disposed about the muscle fiber axis (Rowe, 1974, 1981: Borg and Caulfield, 1980; Purslow, 1989). These collagen bundles again lie in the plane parallel to the muscle fiber surface and their long axes lie at +55° and −55° to the muscle fiber direction at muscle rest length. The orientation of each of the two crossed-plies of collagen fibers and the crimp angle varies systematically with muscle sarcomere length (Purslow, 1989). Gillies and Lieber (2011) are of the opinion that it is not known if perimysium forms a continuous network across the width of a muscle and from origin to insertion of fascicles, whereas the micrographs from studies on a range of muscles from rats, rabbits, sheep, pigs, cattle, and chickens appear to demonstrate quite clearly that the perimysium does indeed form a continuous network across the muscle fascicle (Rowe, 1981; Nishimura et al., 1994; Purslow and Trotter, 1994; Liu et al., 1995; Passerieux et al., 2007). A considerable amount of experience has been accumulated in our laboratory in dissecting out large sheets of perimysium from the bovine semitendinosus muscle for mechanical testing and thermal analysis in a series of publications ranging from 1989 (Lewis and Purslow, 1989) up until the present day (Latorre et al., 2019), which leads this author to the conclusion that perimysium does indeed form a continuous network across the width of a muscle and from origin to insertion of fascicles. It is also clear that the thickness and spatial distribution of perimysium varies greatly between different muscles, as shown in Figure 2 for three bovine muscles (Purslow, 1999). It should also be noted that the resilient protein elastin is present in small amounts in the perimysium of most muscles but that the amount of elastin is increased dramatically in muscles such as bovine latissimus dorsi and semitendinosus (Bendall, 1967), where it is thought to act as an elastic energy store. Rowe (1986) showed that elastin fibers were predominantly associated with the perimysium and epimysium of bovine semitendinosus and longissimus dorsi muscles.

### Structure of the Epimysium

The epimysium is a thick connective tissue layer that is composed of coarse collagen fibers in a proteoglycan matrix. The epimysium surrounds the entire muscle and defines its volume. The arrangement of collagen fibers in the epimysium varies between muscles of different shapes and functions. For instance, the collagen fibers in the relatively thin epimysium of the long strap-like M. sternomandibularis in the cow has two sets of collagen fibers running at ± 55 to the muscle long axis (Purslow, 2010), whereas the collagen fibers in the thicker epimysium of M. semitendinosus in the same animal are close-packed and parallel to the muscle long axis, and merge into the tendon. The thicker epimysium of pennate muscles form a sheet-like aponeurosis that acts as a wide base of muscle attachment (Sakamoto, 1996).

### Continuity of IMCT, Tendons and Deep Fascia

**FIGURE 3:**
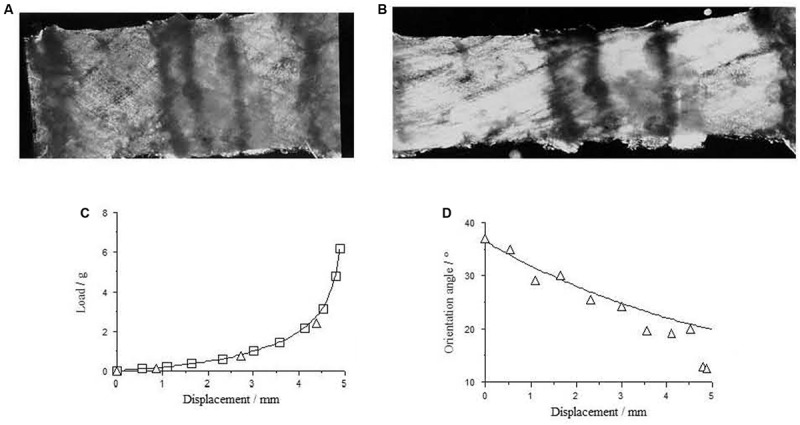
Perimysium excised form bovine semitendinosus muscle 24 h post-mortem **(A)** upstretched and **(B)** stretched transverse to the muscle fiber direction, with the resulting load-deformation curve shown in **(C)**. In **(D)** measurements of the angle between the collagen fiber bundles and the stretching direction (triangles) are a reasonable fit to a model sf strain-induced reorientation (Purslow, 1989; fitted line) in the perimysium. Reproduced from Purslow (1999).

Although the various IMCT structures are often described as sheaths that separate individual fibers (endomysium) fascicles (perimysium) and whole muscles (epimysium), in reality these structures form continuous networks that connect and coordinate the muscle elements within them. The endomysium clearly forms a continuous network structure within a fascicle and perimysium clearly forms another continuous network within the whole muscle As the perimysium approaches the surface of the muscle it merges seamlessly with the epimysium (Turrina et al., 2013). At the ends of the muscle, the epimysium thickens and merges with the tendons (Benjamin, 2009). Tendinous connections from several muscles onto the deep fascia of limb muscles have been observed, and it appears that these connections provide myofascial continuity between the different muscles of the limb (Stecco et al., 2007). It is hypothesized that this continuity of connections between IMCT and fascia coordinate the action of agonistic muscles. Within this hierarchy of connections, the nature of connections between the endomysial and perimysial networks at the surface of muscle fascicles. is less well defined. Rowe (1981) observed an open network of fine wavy collagen fibers joining the thick, dense planar network of collagen fiber bundles in the perimysium to the endomysium of muscle fibers at the surface of a muscle fascicle. Passerieux et al. (2006) similarly reported connections between the perimysium and the endomysium of muscle fibers at the surface of fascicles in bovine flexor carpi radialis muscle that they termed perimysial junction plates (PJPs). These periodic junctions are formed by branching collagen fiber bundles from the perimysium inserting into the surface of the endomysium. Gillies and Lieber (2011) show some evidence of similar connections in their scanning electron micrographs of preparations from mouse extensor digitorum longus muscles. PJPs are staggered at the surface of each muscle fiber and separated by a distance of approximately 300 μm. Transmission electron microscopy and immunohistochemistry studies of PJPs have revealed a concentration of muscle fiber nuclei and mitochondria in the muscle fiber underneath the PJP (Passerieux et al., 2006). This suggests that PJP’s may be a point of transmission of mechanical information and stimuli into the muscle fibers which affects expression in the cell (i.e., points of mechanotransduction). A point of debate is whether these junctions can also function as pathways for transmission of active and passive forces. The preparation of specimens for Figure 3 involved the fracture of freeze-dried fixed samples, and Passerieux et al. (2006) noted that fracture removed the dense layer of perimysium from the surface of the fascicle, leaving only the perimysial collagen strands attached to PJPs. They argue that this means that these junctions are very strong. However, these connections are only sporadic (more than 100 sarcomeres apart) and mechanical tests (Lewis and Purslow, 1990) showed that the breaking strength of endomysial junctions was considerably below the strength of the perimysial network, indicating that these junctions may not be strong enough to transmit large forces.

The main components of IMCT are the fibrous collagen types I and III in a matrix of proteoglycans, with the non-fibrous type IV present in the basement membrane of the muscle cells. Small amounts of fibrous type V collagen and several of the fiber-associated collagens are also present. The endomysium and perimysium have distinct proteoglycan and collagen compositions, as detailed by Purslow and Duance (1990). Light and Champion (1984) estimated that type III made up 16% of the total (type I + III) in epimysium, 28% in the perimysium and 62% of the endomysium in bovine pectoralis muscle. These proportions vary between the IMCT of different muscles. Comparing six bovine muscles, Light et al. (1985) reported type III contents in the range of 14–30% in the epimysium, 25–43% in the perimysium and a much smaller variation of 53–58% in the endomysium.

## Physiological Roles of IMCT

Intramuscular connective tissue has a wide range of functions. At the most mundane level, it organizes and carries the neurons and capillaries that service each muscle cell. Especially at the level of the perimysium, it provides the location of intramuscular deposits of fat. It patterns muscle development and innervation, as proliferation and growth of muscle cells is stimulated and guided by cell–matrix interactions. These roles have been discussed previously (Purslow, 2002). This review focuses on the current knowledge of the mechanical roles of IMCT in relation to the transmission of contractile force, passive tension in stretched muscle, and the adaptation of muscle due to mechanotransduction.

Hill (1949) stated that the mechanical properties of skeletal muscle could be described by a contractile element (CE), an elastic element in series with this (SE) and another Eleatic component in parallel to the CE and SE. He was at pains to point out that this was a mechanical description only, and that it was not necessary to identify the structures responsible for the SE and PE response. By “elastic” he meant that these elements would return to their original dimensions after loading, and so act as spring-like stores of strain energy. He noted that the stress-strain behavior of his hypothetical SE and PE elements would be non-linear, in that their stiffness would increase with strain. This “Hill-type three element model” conditioned many discussions of the mechanical behavior of muscle subsequently. This model essentially focuses on the contribution of each “element” in tension, a constraint that is discussed below.

### Experimental Data

Two papers published in the mid 1980’s characterize two very different streams of thought about the contribution of IMCT to the mechanical functioning of muscle. Magid and Law (1985) measured the passive load-extension behavior of single fibers from frog muscle with and without surrounding endomysium and concluded that this IMCT structure contributed very little to the passive tension of muscle. This finding generated a focus on titin as the principal intracellular structure responsible for passive elasticity in the tissue. Looking at Figure 1E, it is clear that dissection of a single muscle fiber with some surrounding endomysium must involve the disruption of the continuous network structure of the endomysium, a process that is much easier to achieve without damaging the muscle fiber in frog muscle than in mammalian species, hence frog muscle being the material of choice for single fiber physiology studies. It is more difficult, but possible, to isolate viable single fibers from mouse extensor digitorum longus muscle and measure their properties (Lännergren and Westerblad, 1987, 1991; Westerblad et al., 1993). Measurements of the passive elasticity of muscle by tensile tests on single fibers have also been used to look at changes in IMCT in diseased human muscle (Mathewson et al., 2014) or changes due to unloading on rat soleus fibers (Toursel et al., 2002). Meyer and Lieber (2018) made a direct comparison of the passive elasticity of mouse versus frog single fibers using the same apparatus and protocol, and showed that endomysium had a greater contribution to passive elasticity in the mouse muscle fibers than those from the frog. By testing the tensile properties of whatever remnant of the endomysial network that clings to the surface of an isolated single fiber, these investigations unequivocally demonstrate the non-linear tensile properties of the endomysium. The thinking behind this line of investigation is very much in accord with the three-element Hill-type model of muscle mechanics that distinguishes a contractile element, a series-elastic element and a parallel elastic element. By equating the endomysium with the parallel elastic element, this type of analysis forces our thinking into consideration of the tensile properties of the endomysium. Purslow and Trotter (1994) studied changes in the orientation and waviness of collagen fibers in the endomysial network layer with muscle sarcomere length and found that endomysium is non-linearly elastic but extremely compliant in tension over the physiological range of sarcomere lengths. A similar investigation with perimysium also showed that this network of crimped collagen bundles is very compliant in tension over the range of physiologically relevant sarcomere lengths (Purslow, 1989). By constraining thought to tensile properties of these planar fibrous networks of wavy collagen fibers or fiber bundles, it was concluded that IMCT structures are too compliant in tension to efficiently contribute to the transmission of contractile force at the sarcomere lengths where muscle generates most force. However, as noted above, this conclusion is simply based on the assumption that these ECM structures are working in tension. As discussed below, that is now thought to be incorrect, and that their through-plane shear properties are more important. Using finite element models based upon the Hill-type three-element model, Marcucci et al. (2019) have recently suggested that the contribution to the parallel elastic component in passive muscle elasticity may nevertheless be substantial.

The second stream of thought about the functioning of connective tissue within muscle was generated by the observation by Street (1983) that short segments of myofibrils from adjacent fibers adhering to the endomysium of a single fiber dissected from frog semitendinosus muscle changed length when the intact fiber was stretched. This gave birth to the idea that forces are transferred laterally between adjacent fibers by shear through the endomysium. This mechanism explains why length changes in non-activated muscle fibers follow the length changes of actively contracting neighboring fibers when only a subset of muscle motor units are activated in sub-maximal contraction.

A general recognition that force transmission can readily occur between adjacent muscle fibers has been followed by evidence that myofascial force transmission can occur between fascicles even between adjacent muscles, as summarized by Huijing (2009); Maas and Sandercock (2010), and Maas (2019). While there is some dispute that epimysial force transfer between individual muscles is significant (Diong et al., 2019), the general idea of lateral force transmission between adjacent fibers within a muscle fascicle is less controversial. However, this concept requires a change of mental picture about the functional properties of IMCT is two ways. Firstly, although it is possible to discern the tensile properties of endomysium by comparing the tensile properties of skinned single muscle fibers to muscle fibers with endomysium, or small groups of fibers with a part of intact endomysial network between them, the relevance of this needs to be rethought. If a prime function of the endomysium is to coordinate strains between adjacent muscle fibers and keep sarcomeres in register with each other by transmission of forces by shear, does measurement of tensile properties really help to understand these important shear properties? Secondly, from a materials science or biophysical view of endomysium and perimysium, it is natural to focus on the tensile, in-plane properties of a planar fibrous network and the non-linear behavior that these exhibit due to strain induced reorientation and de-crimping. This was the approach originally applied to analysis of the perimysium (Purslow, 1989) and to the endomysium (Purslow and Trotter, 1994), and in both cases the result was only to highlight the worryingly high compliance of these networks in tension. It should be remembered that the endomysium studied by Purslow and Trotter (1994) was from a very obviously series-fibred muscle (bovine sternomandibularis muscle) where the none of the intrafascicularly terminating, short muscle fibers run the length of a fascicle and the great majority have no myotendinous attachment, so that transmission of contractile force via the endomysium is the only option. We should also note that the endomysial connections between intrafascicularly terminating fibers in series-fibred muscles are essentially acting as part of the series elastic component in Hill’s three-element model; efficient transfer of force from the contractile element out to tendons and eventually bones requires a series-elastic “link” that (a) does not dissipate energy (which would waste the energy of contraction) but stores it elastically, and (b) is relatively stiff, as a very stretchy or compliant linkage would not efficiently translate muscle contractions into movement of the bones. Analysis of tensile properties of these planar collagenous networks continues to be a common mindset (e.g., Bleiler et al., 2019). The highly compliant tensile properties of endomysium provide little resistance to the longitudinal and circumferential dimensional changes in working muscle fibers; the endomysium easily allows and follows changes in fiber geometry as muscle contracts and is passively lengthened. This, however, is not inconsistent with providing a reasonably efficient transmission of force by translaminar shear (shear through its thickness).

Tensile tests on small sheets of perimysium isolated by careful dissection from muscle are possible and show the obvious non-linear stress-strain behavior expected of a compliant network that suffers reorientation at finite strains and a straightening of initially wavy or crimped collagen fiber bundles. An example if given in Figure 3. Tensile tests on isolated perimysium from large bovine muscles have continued to be performed only because of the relevance of these properties to the textural properties of muscle eaten as meat (e.g., Latorre et al., 2019), but of course do not shed light on the functioning of this IMCT structure *in vivo*, except to reinforce the point that perimysium, like endomysium, is easily deformed in tension at resting muscle lengths.

It is clear that the majority of muscles undergo shape changes as they contract (Dick and Wakeling, 2017, 2018; Roberts et al., 2019); fusiform muscles bulge in mid-section as they contact, as do fan-shape muscle such as the pectoralis and all unipennate, bipennate and multipennate muscles. This sounds like a trivial observation, but consideration of how a fibrous composite tissue can change shape reveals that, in order to do so, some elements in the tissue as a whole must be allowed to shear past neighboring elements. The question is; which elements, at what scale of structure? If the endomysium is tightly coordinating forces and displacements between adjacent muscle fibers in a fascicle, then the likelihood is that shear displacements could be accommodated between fascicles. In a crude experiment, Purslow (2002) demonstrated that shape changes caused by manipulation bovine semitendinosus muscle in rigor produced slippage between fascicles, but not within fascicles. Schmalbruch (1985) also discusses this mechanism. The shear strains within different muscles are substantial and vary between diverse muscles (Mutch, 2015).

It has been postulated that variations in the size and shape of fascicles, and therefore in the spatial distribution of perimysium, was related to variations in the shear strains that need to be accommodated in differently shaped muscles as they contract (Purslow, 2002, 2010). This idea has since been supported by computational models (see below) and argues that shear stains in the perimysium must be larger than shear strains though the endomysium between muscle fibers in a fascicle. This is in contrast to the interpretation of those researchers (e.g., Huijing, 2009; Maas, 2019) who stress the importance of lateral force transmission between fascicles and between entire muscles (epifascial force transmission) as an important physiological function, as a perimysium easily deformed in shear would not be an efficient means to transmit contractile force laterally between fascicles.

### Modeling Stress Transfer Between Muscle Fibers by Shear

**FIGURE 4:**
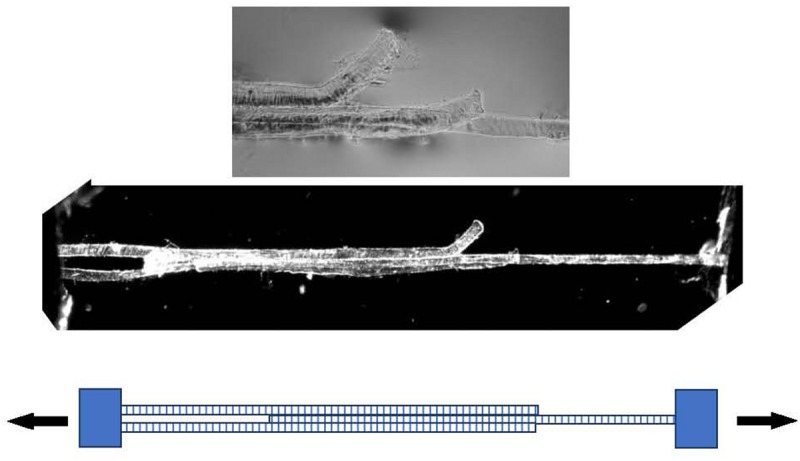
Three muscle fibers dissected post-rigor from rat gastrocnemius muscle forming a Y-shaped specimen suitable for measuring the shear properties of endomysium. Bottom: schematic representation. Middle panel: polarized light micrograph of the whole preparation. Top panel: higher-magnification phase contrast image of the mid-section, showing the free ends of the outer two fibers.

Models of the shear properties of perimysium and endomysium at different sarcomere lengths (Purslow, 2002; Appendix A) led to the idea that shear transmission of force could be relatively efficient over a wide range of sarcomere lengths. These simple ideas based on fibrous composites theory were developed using estimated ranges of shear parameters, as no measured values were available. In developing these models of translaminar shear through a reorientating planar network of near-random, wavy collagen fibrils, Purslow (2002) reasons that in practical terms the shear modulus of the endomysium would be relatively insensitive to changes in sarcomere length. These models have not been tested, because of the difficulty in making experimental observations. It has been proposed to use an overlapping shear configuration of three muscle fibers (as shown in Figure 4) to directly measure the shear stiffness of the endomysium. These experiments have not yet been performed in the author’s laboratory due to a lack of the strain vector mapping necessary to extract shear displacements in the mid region of the specimen. The specimen is shown only to demonstrate the viability of such preparations.

### Computational Models of Stress Transfer in Muscle by Shear

Various computational models (principally finite elements (FE) models) have been used to explore the possible role of IMCT in the active and passive mechanical properties of muscles. These models are very attractive in that they can represent the complex three-dimensional architecture of the tissue at various levels. A full anisotropic set of (non-linear) moduli (both extensional and shear) and the anisotropic poisons ratios for both muscle fiber elements and IMCT elements, together with a detailed representation of their shape and spatial distribution would be an ideal starting point for building such complex computational models. However, a full set of extensional, shear and poison’s ratio parameters for muscle fibers and either the endomysium of perimysium, or both, is not available. So, while a great number of parameters can be estimated from experimental studies, inevitably there needs to be some assumed range of values for many of the parameters, not least the shear properties of the IMCT components (both for shear through the thickness along the muscle fiber direction and transverse to it). Thus, Yucesoy et al. (2002) used a non-linear strain energy density function to model the IMCT in their FE model of interactions between muscle fibers and endomysium that contained different extensional stiffness constants along and across the muscle direction but equal shear stiffness in all directions. This Linked Fiber-Matrix Mesh model approach has been discussed further by Yucesoy and Huijing (2012) and applied to a study of muscle paralysis by botulinum toxin (Turkoglu and Yucesoy, 2016). Sharafi and Blemker (2011) were very explicit (in their table 1) of the sources of the range of parameters that they included in their FE model of force transmission from intrafascicularly terminating muscle fibers. They noted that shear moduli for the endomysium had not been measured and used estimated values for an (isotropic) shear modulus in the range 3.7–5 kPa. In their geometrically simplified FE model of a single cylindrical muscle fibers embedded in an endomysial matrix, Zhang and Gao (2012) use a range of mechanical property parameters from previous models, including that of Sharafi and Blemker (2011). They assume a shear modulus of 0.3–0.6 kPa along the muscle fiber direction and 0.15–0.3 transverse to the muscle fiber direction. These three FE models were are all very useful in exploring the patterns of complex forces and deformations in muscle as a composite tissue and, not withstanding the differences in assumed shear parameters and other differences in the model assumptions and constraints, all three produced results which indicated a strong role for transmission of force between fiber and ECM or between adjacent fibers by shear. Sharafi and Blemker (2010) also used the FE model approach to examine the effects of fascicle shape and size (and therefore perimysial spatial distribution) on the properties of muscle. They noted that measured values of shear moduli for muscle fibers, fascicles and IMCT did not exist, and were likely to vary considerably from muscle to muscle, so explored a wide range of these parameters in their model. Using fascicle size and shape data from two muscles (rabbit rectus femoris and soleus), Sharafi and Blemker (2010) showed that perimysial thickness, the spatial staggering of fascicles to each other and the anisometric ratio of the fascicle were most important in determining the overall macroscopic mechanical properties of each muscle. Over a range of mechanical parameters studied, their simulations predicted higher shear strains in the perimysium than within the fascicles (i.e., in muscle fibers or the endomysium) and supported the concept that variations in the spatial distribution of perimysium in a muscle, defining muscle fascicles of varying sizes and shapes, generally follow the need to accommodate more of less shear strains in a given plane depending on the shape changes and ranges of motions necessary for different muscles to fulfill their different functions. This idea is echoed by Mutch (2015) who supposes that fascicles are smaller, and separated by thinner perimysium, in long strap-like muscles such as bovine sternomandibularis or human sartorius because these contraction of these muscles produce only small shear displacements within them, compared to the bigger fascicles and thicker perimysium in some pennate muscles, where shear displacements are greater. From a range of ultrasonic imaging studies on human muscles *in vivo*, Purslow (2002) in his table 2 presented data to show that maximum shear strains between fascicles in pennate muscles could be as high as 2.05, and could vary substantially between the muscles studied.

While actual measurements of the shear properties of endomysium through experiments such as those proposed above in relation to Figure 4 may provide accurate data to feed into such computational models, it is this author’s view that the conclusions form FE models such as those cited here would not be affected greatly, if at all. Over a wide range of simulated mechanical parameters, these models form a consensus view that shear transmission of force through a relatively stiff endomysium is an important feature of muscle function, whereas the more shear-compliant perimysium provides a mechanism for fascicles to shear past each other, permitting the macroscopic shape changes necessary for muscles to change shape as they contract.

Supersonic shear imaging (SSI) studies have provided estimates of the macroscopic shear modulus of muscles *in vivo*. Lacourpaille et al. (2012) measured values of shear modulus in the range of 2.99–4.5 kPa in nine resting muscles of human subjects. Nakamura et al. (2016) reported increasing values of 8.1–41.6 kPa for human gastrocnemius with increasing passive ankle dorsiflexion. In their review, Lima et al. (2018) cite values from a range of studies on different muscles ranging from 7 to 70 kPa depending on degree of muscle lengthening and joint rotations and note that values increase with increasing muscle activity. Chakouch et al. (2015) used the different technique of magnetic resonance elastography (MRE) to measure the shear modulus along nine muscles in the human thigh at rest and report values ranging from 3.91 to 6.15 kPa. SSI and MRE both use high frequency waves to study the tissue. Because of the frequency-dependent viscoelastic nature of muscle and IMCT, the shear moduli measured in SSI and MRE are likely to be high estimates. However, these data do give values for macroscopic shear properties of muscle that could be used as end-point comparisons from computational models. It should be emphasized that these reported shear moduli are for the whole muscle tissue, not the IMCT. It is clear that these shear moduli vary between active and passive muscle and increase with increasing muscle length or displacement. It is reasonable to speculate that the translaminar shear moduli of the perimysium and endomysium may also change with muscle length, as changes in the orientation of collagen fibers with the IMCT network with changing sarcomere length have been reported for both perimysium (Purslow, 1989) and endomysium (Purslow and Trotter, 1994). Simple fibrous composite modeling of the effects of collagen fiber reorientation (Purslow, 2002) do predict that, over the entire range of sarcomere lengths that can be achieved in muscle, the translaminar shear modulus in the longitudinal direction will become greater at very long sarcomere lengths, and the translaminar shear modulus in the circumferential direction will become greater at very short sarcomere lengths. However, near muscle rest length the two translaminar moduli are predicted from this simple model (Purslow, 2002) to be more nearly equal and to only change to a very small degree within the normal physiological range of sarcomere length changes. As noted above, this led Purslow (2002) to hypothesize that the shear modulus of the endomysium would be relatively insensitive to changes in sarcomere length. This argument can be equally applied to the shar properties of the perimysium. However, this hypothesis is yet to be confirmed by direct measurements of the shear properties of these IMCT networks.

Pamuk et al. (2016) combined magnetic resonance and diffusion tensor imaging methods to assess deformation along the muscle fiber direction in human medial gastrocnemius muscle *in vivo*, including shear strains. As well as noting a great inhomogeneity in longitudinal strains between muscle fibers, Pamuk et al. (2016) observe that analysis of along-fiber shear strains confirm that there is a great amount of shear displacement between muscle fascicles during *in vivo* movements of this muscle.

If the shear properties of the endomysium are designed to keep adjacent muscle fibers closely aligned and coordinated with efficient force transfer between them, whereas the shear properties of the perimysium are designed to facilitate large shear strains in a working muscle, then it follows that (a) computational models should not use the same estimates of shear properties for both endomysium and perimysium, and (b) the mechanisms for adaptive growth or degradation of endomysium and perimysium may be differently regulated, or may respond to different ranges of stimuli.

## Changes of IMCT in Muscle Adaptation

Part of the adaptation of muscle to exercise, disuse and overload injury involves changes to the IMCT (Kjaer, 2004; Mackey et al., 2011, 2017; Hyldahl et al., 2015). There are also some changes in the IMCT content of muscles as animals and people grow and age, as reviewed by Kragstrup et al. (2011). Post natal muscle growth is generally by muscle fiber hypertrophy, which means that, if the thickness of endomysial and perimysial structures remains constant, there would be a decreased intramuscular collagen concentration as the volume of the muscle cells increased. In the rapid growth of muscle in young farm animals, there is no obvious change in collagen concentration (McCormick, 1994), arguing that the volume fraction of muscle occupied by endomysial and perimysial IMCT structures keeps pace with the growth in muscle fiber volume. Kragstrup et al. (2011) review the complicated evidence from both animal and human studies indicating that, after maturity is reached, there is a increase in intramuscular collagen content, as well as change in the properties of IMCT due to age-related covalent crosslinking. In the study of advanced human aging, it is unclear if an increase in IMCT concentration is simply a function of muscle fiber volume loss due to inactivity. It is also clear that the connections within muscle tissue afforded by IMCT and myotendinous joint connections are predominant sites of lesions in muscle strain injuries, as highlighted in a recent meta-analysis (Wilke et al., 2019).

Maintenance and regeneration of IMCT is a balance of synthesis by fibroblasts versus degradation. Matrix metalloproteinases (MMPs) are the principal proteolytic enzymes of IMCT together with the family of metalloendopeptidases described as a disintegrin and metalloproteinase (ADAMs), which act as sheddases, cleaving off the extracellular portions of integrins at the muscle cell surface (Christensen and Purslow, 2016). In their studies of rat soleus muscle, Cha and Purslow (2010a) demonstrated that MMP activity occurs at the endomysium and perimysium, but that the muscle fibers themselves produce large quantities of MMPs. When subject to biaxial stretching a stronger increase in MMP activity is produced by myoblasts than fibroblasts (Cha and Purslow, 2010b). In the response of muscle to overload injury it is particularly evident that connections between muscle cells and IMCT are broken down before remodeling occurs (Mackey et al., 2011; Hyldahl et al., 2015).

Myoblasts and myotubes can produce their own basement membrane collagens (Bailey et al., 1979; Kühl et al., 1982), normally the type I and III collagen fibrous structures of the interstitial region of the endomysium and of the perimysium are considered to be primarily produced by fibroblasts (Chapman et al., 2016). However, this very much depends on the stage of development and conditions within the muscle (such as muscle injury, disease or chronic inflammation). Myoblasts produce type I collagen but this production normally decreases substantially when the myoblasts differentiate into myotubes whereas satellite cells produce type I and III collagens (Alexakis et al., 2007). In contrast, in the mdx mouse, a model for Duchenne muscular dystrophy (DMD), the satellite cells in older muscle can still produce type III collagen but cease to produce type I collagen (Alexakis et al., 2007). Substantial amounts of type I collegen, produced by non-myogenic cells, are present in the fibrosis evident in DMD. Transforming growth factor β (TGF-β) is elevated in muscle injury and also in pathological conditions such as DMD. TGF-β is a well-known regulator of muscle fiber size but also causes elevated collagen type I expression from both myoblasts and differentiated myotubes in cell culture (Hillege et al., 2020). Myogenic progenitor cells arising from satellite cells in growing or repairing muscle form part of the regulation of synthesis of new collagen in the IMCT by fibroblasts. Satellite cells activated by mechanical overload proliferate and secrete exosomes containing microRNAs (MiRNAs) into the ECM. Binding of MiRNAs, and specifically MiR-206, to fibroblasts suppresses collagen synthesis by its inhibiting action on Ribosome-binding protein 1 (Fry et al., 2017). In the absence of satellite cells, or upon their depletion in chronic overload or disease, the lack of exosome-mediated regulation of collagen synthesis in fibroblasts leads to fibrosis. There is therefore evidence to suggest that both collagen synthesis in the endomysium and its degradation by MMPs may be regulated by muscle cells and their associated satellite cells. It is less obvious that these mechanisms are relevant to the control of collagen deposition and degradation in the perimysium, which is not in contact with the muscle cells.

While there has been assumption that high rates of muscle growth must be accompanied by a high rate of turnover (degradation and resynthesis) of IMCT (Etherington, 1987), investigations of collagen metabolism resulting from manipulations of animal nutrition fail to reliably back this up. McCormick (1989) summarized a number of studies on this and concluded that the processes controlling collagen synthesis, degradation and stabilization via covalent crosslinking in response to high or low growth rate are far from clear, based on the fact that some dietary manipulations producing high rates of muscle accretion are accompanied by more intramuscular collagen with a higher solubility (which is presumed by Rompala and Jones (1984) to indicate newly synthesized collagen) whereas some others do not. If collagen solubility is indeed a reasonable measure of newly synthesized collagen, it difficult to explain increased solubility of collagen in the muscle of pigs on a restricted diet, with low growth rate (Kristensen et al., 2002; Therkildsen et al., 2002).

Muscle immobilization also results in changes in IMCT, and these responses may also shed light on its role. Jozsa et al. (1988) reported that the collagen content of soleus, gastrocnemius and tibialis anterior muscles of rats increased when their hind limbs wee immobilized. This was partially a result of loss of muscle fiber volume, but an increase in endomysial and perimysial thickness was also noted. Järvinen et al. (2002) noted similar patterns of fibrotic depositions in both the endomysium and perimysium in the same three muscles of the rat hind limb upon immobilization. Excessive deposition of ECM (fibrosis) js a well-know feature of muscular dystrophies, myopathies and severe muscle injuries, as reviewed by Mahdy (2019). It may be that a fibrotic deposition of IMCT is a mechanism to prevent over-extension and catastrophic rupture of weakened muscle fibers and fascicles. However, the appearance of myofibroblasts as opposed to fibroblasts at sites of connective tissue injury and muscle injury (Li and Huard, 2002; Contreras et al., 2016) complicates the issue and suggests that fibrosis may be a response of a differentiated cell type with a different set of response conditions than normal fibroblasts.

### Mechanotransduction Between Muscle Cells and IMCT; Which Mechanical Signals Matter, and Is It a Two-Way Street?

Mechanotransduction refers to the signally pathways by which cells sense and respond to mechanical stimuli by changes in their expression. Mechanotransduction in muscle is a well-understood concept that has been reviewed extensively (Hornberger and Esser, 2004; Burkholder, 2007; Olsen et al., 2019). It is generally recognized to involve connections from the ECM via integrins (Boppart et al., 2006) with intracellular signaling in large part via MAP Kinases (Martineau and Gardiner, 2001). Constantin (2014) reviews evidence that the dystroglycan complex is another important transmembrane connection involved in muscle mechanotransduction. Other intracellular signaling pathways involved in the response of muscle cells to external mechanical signals include calcium channels (Damm and Egli, 2014) and Yes-associated protein (YAP) activated pathways (Fischer et al., 2016). Nitric oxide synthase also participates as a regulatory mechanism of protein synthesis and degradation in skeletal muscle (Shenkman et al., 2015). However, the IGF-1-Akt-mTOR is thought to be the main positive regulator of skeletal muscle size (Schiaffino et al., 2013).

From the viewpoint of regulating and adapting both muscle fiber volume and properties and IMCT structures to functional demands on muscles, there are two aspects of current muscle mechanotransduction research worth noting. Firstly, while the exact nature of the intracellular signaling pathways and resulting changes in expression have been studied with great precision, the exact nature of the mechanical stimuli that stimulates these is generally less well defined. While Burkholder (2007) is careful in his general discussion to distinguish forces from deformations and to make the important distinction between tension and shear, as well as discussing cyclic versus static loading, the primary investigations he reviewed are generally less concerned with the precise type of mechanical signal that elicited the responses in the signaling pathways they studied. Given that shear transmission of force through the endomysium seems to be an important feature of muscle functioning, an emphasis on shear stresses or shear strains as a primary mechanical signal could be an interesting focus in future work. Juffer et al. (2014) showed that myotubes are sensitive to shear stress imposed by fluid flow. Huijing and Jaspers (2005) postulate that shear effects between adjacent muscle fibers is an important component of the mechanical signals for adaptation. Fischer et al. (2016) stated that regulation of YAP pathways by shear in muscle have not been characterized, but noted that fluid shear stress does activate YAP in osteoblasts and chondrocytes. Meanwhile, information on the activation of the PI3K/AKT/mTOR pathway by mechanical stimulation of cells continues to be obtained by use of cyclic biaxial membrane stretching as a stimulus (Da et al., 2020) where it is hard to dissect the exact mechanical component of the stimulus. Although the activation of the mTOR pathway by elevated IGF-1 is a principal mechanism in the regulation of muscle fiber size, it should be noted that a variety of other factors activate mTOR, including reduced myostatin expression (Egerman and Glass, 2014) and nitric oxide synthase (NOS) signaling (Ito et al., 2013), as well as the direct effects of mechanical loading (Hornberger et al., 2006). Mechanical loading of differentiated myotubes by either cyclic biaxial stretching (Wozniak and Anderson, 2009) or pulsating fluid shear stress (Juffer et al., 2014) promotes Nitrous oxide (NO) production.

The second point to note is that most studies of mechanotransduction in muscle are concerned with transfer of mechanical information from the ECM to the muscle cells. But connections between muscle cells and ECM also transmit forces into the ECM. Regulation of IMCT due to mechanical signaling from contractile forces is far less studied. This is an important aspect if we want to know what signals could control the deposition or remodeling of IMCT beyond passive stretching of the muscle. Mechanical signals for IMCT deposition or degradation can reasonably be expected to be either strains or stresses experienced by fibroblasts, and indeed collagen and proteoglycan synthesis is primarily the function of these cells. Our current understanding of muscle function envisages relatively small shear strains within a fascicle, i.e., efficient force transmission through an endomysium with a relatively high shear stiffness, whereas perimysial boundaries allow large shear strains between fascicles. Given these differences, it is not unreasonable to suggest that the amplitude or nature of the signals controlling the growth or degradation of these two different IMCT structures are likely to be different. Miller et al. (2015) demonstrated an increase in the rate of collagen synthesis within human gastrocnemius muscle after an acute bout of strenuous exercise in the same timeframe as increased myofibrillar protein synthesis, indicating a coordination of response from both myocytes and fibroblasts. However, it was not possible in this study to distinguish between synthesis of collagen in the endomysium versus the perimysium. It is also possible that some of the collagen synthesis was related to micro-injury in response to overload and the consequent macrophage infiltration and TGF-β upregulation of fibroblast activity seen in muscle injury repair (Kim and Lee, 2017). While mechanisms of mechanotransduction in fibroblasts generally are known (Chiquet et al., 2003, 2007), fibroblasts in different tissues have different responses (Mackley et al., 2006) and fibroblasts isolated from different muscles in the same animals are known to have different expression (Archile-Contreras et al., 2010). By definition, fibroblasts synthesizing components of endomysium have a different expression than fibroblasts synthesizing perimysial components in the same muscle.

The control of degradation by MMPs may also be via signaling to fibroblasts, although it is more likely that signaling within muscle cells also has a large contribution to MMP expression and activity, as discussed above. Any study of the effects of stimuli on the production of MMPs by muscle cells is complicated by the fact that MMPs function as intracellular signaling molecules as well as extracellular proteases (Mannello and Medda, 2012). A perinuclear concentration of MMP activity has been observed in myoblasts (Cha and Purslow, 2010a) and in differentiated myotubes (Purslow et al., 2012).

As discussed above, the tight linkage of mechanotransduction at the muscle cell-ECM interface focuses attention on endomysial-muscle cell interactions, but the perimysium is only sporadically connected to the endomysium of muscle cells at PJPs. While the mechanotransduction pathway afforded by PJP structures (with their concentrations of sub sarcolemmal myonuclei) may be pass external mechanical information into the muscle cells, it is more difficult to postulate that the muscle cells also regulate turnover of the perimysium, unless perimysial fibroblasts are differentiated from endomysial fibroblasts and respond differently to signals coming from the muscle cells so as to synthesize and degrade this separate and distinct IMCT structure separately.

A final consideration on the control of IMCT turnover and deposition centers on the scaling of stresses at muscle fiber surface with respect to muscle fiber growth. A normal assumption is that the force produced by a fiber will increase in proportion to its cross-sectional area, i.e., radius squared, on the basis that the number of myofibrils per muscle cell should scale with CSA. As the muscle fiber contracts, the generated surface shear stress would be proportional to the force divided by the surface area of the fiber (which is a linear function of radius). This would imply that, as a fiber grows in radius through hypertrophy, for a given force output per myofibril, the shear stress at the surface of the fiber would be increasing. Above a limiting value, increased signaling to the muscle cell could then trigger remodeling by release of MMPs/ADAMs, and paracrine signaling from muscle cells to fibroblasts could affect collagen synthesis. At least in cardiac muscle, the sheddase activity of ADAMS is thought to reduce integrin-mediated signaling with the ECM during hypertrophy (Manso et al., 2006). An increase in the thickness of the endomysium will lower the shear strains in it back to some value below this triggering value. However, the assumption that muscle hypertrophy proportionally increases the number of myofibrils in the cross-section of growing muscle fibers, increasing the force the produce in relation to the square of the muscle fiber radius, has been called into question (Haun et al., 2019). Krivickas et al. (2011) demonstrate that contractile force generated in single muscle fibers is a linear function of their diameter (or radius). If this analysis is true, then there would be no change in the magnitude of shear stresses on the fiber surface of muscle fibers grow (as shear stress to contractile stress is a constant ratio of both contractile stress and surface shear stress scales linearly with radius). However, force measurements with intact (non-skinned) muscle fibers from the iliofibularis muscles of Xenopus larvae clearly show that the fiber cross-sectional area is proportionately related to the forces they can generate (Jaspers et al., 2008). This issue could be resolved by a clearer understanding of the shear properties of the endomysial- muscle fiber interface and the cellular responses of both muscle cells and fibroblasts specifically to interfacial shear stimuli.

## Conclusion

Our understanding of the role of IMCT in normal muscle functioning and its role in muscle adaptation and response to injury has undergone considerable revision and is continuing to evolve. It is arguably a legacy of the Hill three-element model that a great deal of thinking on the mechanical roles of IMCT and experimental approaches designed to measure these have centered in the past on tensile properties, in relation to the “passive elastic element.” However, we must remember that the Hill model is just a representation of the macroscopic mechanical behavior of muscle, rather than a mechanistic representation with insight into molecular or structural mechanisms. Although Hill (1949) warned that a three-element model was only a representation of the mechanical behavior of muscle, and that structural elements of the muscle cells and IMCT could not be directly assigned to the SE and PE elements, a mindset of elastic “springs” in series and parallel with the contractile elements has blinkered attention to tensile mechanical behaviors of components. In reality, the in-plane tensile properties of the endomysium and perimysium are very compliant at normal *in vivo* sarcomere lengths, so freely allowing the length changes needed in actively contracting and passively stretching muscle fibers. But, the shear linkages through the thickness of the endomysium keeps adjacent fibers in register and laterally transmits force. While shear deformations in IMCT can be interpreted in terms of its contribution to the effective tensile stiffness of muscle in the muscle fiber direction, the field is moving toward a clear understanding that the shear properties of IMCT networks are different from tensile properties, and most probably the growth and turnover of IMCT structures are sensitive to shear parameters. While measures of tensile properties of endomysium as a passive elastic element in single fiber experiments has provided many insights into muscle properties, it is arguable that what the field needs most is detailed measurements of the translaminar shear properties of the endomysial and perimysial networks.

## Author Contributions

PP is the sole author of this work therefore solely designed, wrote and submitted this manuscript.

## Conflict of Interest

The author declares that the research was conducted in the absence of any commercial or financial relationships that could be construed as a potential conflict of interest.

## References

[B1] Alexakis C. Partridge T. Bou-Gharios G. (2007). Implication of the satellite cell in dystrophic muscle fibrosis: a self-perpetuating mechanism of collagen overproduction. *Am. J. Physiol. Cell Physiol.* 293 C661–C669. 10.1152/ajpcell.00061.2007 17475662

[B2] Archile-Contreras A. C. Mandell I. B. Purslow P. P. (2010). Phenotypic differences in matrix metalloproteinase 2 activity between fibroblasts from 3 bovine muscles. *J. Anim. Sci.* 88 4006–4015. 10.2527/jas.2010-3060 20802142

[B3] Bailey A. Shellswell G. Duance V. (1979). Identification and change of collagen types in differentiating myoblasts and developing chick muscle. *Nature* 278 67–69. 10.1038/278067a0 763352

[B4] Bendall J. R. (1967). The elastin content of various muscles of beef animals. *J. Sci. Food Agricult.* 18 553–558. 10.1002/jsfa.2740181201

[B5] Benjamin M. (2009). The fascia of the limbs and back–a review. *J. Anat.* 214 1–18. 10.1111/j.1469-7580.2008.01011.x 19166469 PMC2667913

[B6] Bleiler C. Castañeda P. P. Röhrle O. (2019). A microstructurally-based, multi-scale, continuum-mechanical model for the passive behaviour of skeletal muscle tissue. *J. Mech. Behav. Biomed. Mater.* 97 171–186. 10.1016/j.jmbbm.2019.05.012 31125890

[B7] Boppart M. D. Burkin D. J. Kaufman S. J. (2006). α7β1-Integrin regulates mechanotransduction and prevents skeletal muscle injury. *Am. J. Physiol. Cell Physiol.* 290 C1660–C1665. 10.1152/ajpcell.00317.2005 16421207

[B8] Borg T. K. Caulfield J. B. (1980). Morphology of connective tissue in skeletal muscle. *Tissue Cell* 12 197–207. 10.1016/0040-8166(80)90061-0 7361300

[B9] Bowman W. (1840). On the minute structure and movements of voluntary muscle. *Philos. Trans. R. Soc. Lond.* 130 457–501. 10.1098/rstl.1840.0022

[B10] Burkholder T. J. (2007). Mechanotransduction in skeletal muscle. *Front. Biosci.* 12:174–191. 10.2741/2057 17127292 PMC2043154

[B11] Cha M. C. Purslow P. P. (2010a). Matrix metalloproteinases are less essential for the in-situ gelatinolytic activity in heart muscle than in skeletal muscle. *Compar. Biochem. Physiol. Part A* 156 518–522. 10.1016/j.cbpa.2010.04.014 20427022

[B12] Cha M. C. Purslow P. P. (2010b). The activities of MMP-9 and total gelatinase respond differently to substrate coating and cyclic mechanical stretching in fibroblasts and myoblasts. *Cell Biol. Int.* 34 587–591. 10.1042/CBI20090096 20218972

[B13] Chakouch M. K. Charleux F. Bensamoun S. F. (2015). Quantifying the elastic property of nine thigh muscles using magnetic resonance elastography. *PLoS One* 10:e0138873 10.1371/journal.pone.0138873 PMC4580449 26397730

[B14] Chapman M. A. Meza R. Lieber R. L. (2016). Skeletal muscle fibroblasts in health and disease. *Differentiation* 92 108–115. 10.1016/j.diff.2016.05.007 27282924 PMC5079803

[B15] Chiquet M. Renedo A. S. Huber F. Flück M. (2003). How do fibroblasts translate mechanical signals into changes in extracellular matrix production? *Matrix Biol.* 22 73–80. 10.1016/S0945-053X(03)00004-0 12714044

[B16] Chiquet M. Tunc-Civelek V. Sarasa-Renedo A. (2007). Gene regulation by mechanotransduction in fibroblasts. *Appl. Physiol. Nutr. Metab.* 32 967–973. 10.1139/H07-053 18059623

[B17] Christensen S. Purslow P. P. (2016). The role of matrix metalloproteinases in muscle and adipose tissue development and meat quality: a review. *Meat Sci.* 119 138–146. 10.1016/j.meatsci.2016.04.025 27180222

[B18] Constantin B. (2014). Dystrophin complex functions as a scaffold for signalling proteins. *Biochim. Biophys. Acta* 1838 635–642. 10.1016/j.bbamem.2013.08.023 24021238

[B19] Contreras O. Rebolledo D. L. Oyarzún J. E. Olguín H. C. Brandan E. (2016). Connective tissue cells expressing fibro/adipogenic progenitor markers increase under chronic damage: relevance in fibroblast-myofibroblast differentiation and skeletal muscle fibrosis. *Cell Tissue Res.* 364 647–660. 10.1007/s00441-015-2343-0 26742767

[B20] Da Y. Mou Y. Wang M. Yuan X. Yan F. Lan W. (2020). Mechanical stress promotes biological functions of C2C12 myoblasts by activating PI3K/AKT/mTOR signaling pathway. *Mole. Med. Rep.* 21 470–477. 10.3892/mmr.2019.10808 31746379

[B21] Damm T. B. Egli M. (2014). Calcium’s role in mechanotransduction during muscle development. *Cell Physiol. Biochem.* 33 249–272. 10.1159/000356667 24525559

[B22] Dick T. J. M. Wakeling J. M. (2017). Shifting gears: dynamic muscle shape changes and force-velocity behavior in the medial gastrocnemius. *J. Appl. Physiol.* 123 1433–1442. 10.1152/japplphysiol.01050.2016 28860176 PMC5814684

[B23] Dick T. J. M. Wakeling J. M. (2018). Geometric models to explore mechanisms of dynamic shape change in skeletal muscle. *R. Soc. Open Sci.* 5:172371. 10.1098/rsos.172371 29892420 PMC5990834

[B24] Diong J. Héroux M. E. Gandevia S. C. Herbert R. D. (2019). Minimal force transmission between human thumb and index finger muscles under passive conditions. *PLoS One* 14:e0212496 10.1371/journal.pone.0212496 PMC6377133 30768639

[B25] Egerman M. A. Glass D. J. (2014). Signaling pathways controlling skeletal muscle mass. *Crit. Rev. Biochem. Mol. Biol.* 49 59–68. 10.3109/10409238.2013.857291 24237131 PMC3913083

[B26] Etherington D. J. (1987). “Collagen and meat quality: effects of conditioning and growth rate,” in *Advances in Meat Research Vol.4. Collagen as a Food*, eds Pearson A. M. Dutson T. R. Bailey A. J. (New York, NY: Van Nostrand Reinhold), 351–360.

[B27] Eyre D. (1987). Collagen cross-linking amino acids. *Methods Enzymol.* 144 115–139. 10.1016/0076-6879(87)44176-1 3626870

[B28] Fischer M. Rikeit P. Knaus P. Coirault C. (2016). YAP-mediated mechanotransduction in skeletal muscle. *Front.Physiol.* 7:41 10.3389/fphys.2016.00041 PMC4754448 26909043

[B29] Fry C. S. Kirby T. J. Kosmac K. McCarthy J. J. Peterson C. A. (2017). Myogenic progenitor cells control extracellular matrix production by fibroblasts during skeletal muscle hypertrophy. *Cell Stem Cell* 20 56–69. 10.1016/j.stem.2016.09.010 27840022 PMC5218963

[B30] Gao Y. Kostrominova T. Y. Faulkner J. A. Wineman A. S. (2008). Age-related changes in the mechanical properties of the epimysium in skeletal muscles of rats. *J. Biomech.* 41 465–469. 10.1016/j.jbiomech.2007.09.021 18031752 PMC2248272

[B31] Gaunt A. S. Gans C. (1993). Variations in the distribution of motor end-plates in the avian pectoralis. *J. Morphol.* 215 65–88. 10.1002/jmor.1052150105 29865426

[B32] Gillies A. R. Lieber R. L. (2011). Structure and function of the skeletal muscle extracellular matrix. *Muscle Nerve* 44 318–331. 10.1002/mus.22094 21949456 PMC3177172

[B33] Haun C. T. Vann C. G. Roberts B. M. Vigotsky A. D. Schoenfeld B. J. Roberts M. D. (2019). A critical evaluation of the biological construct skeletal muscle hypertrophy: size matters but so does the measurement. *Front. Physiol.* 10:247 10.3389/fphys.2019.00247 PMC6423469 30930796

[B34] Hijikata T. Ishikawa H. (1997). Functional morphology of serially linked skeletal muscle fibers. *Cells Tissues Organs* 159 99–107. 10.1159/000147972 9575360

[B35] Hill A. V. (1949). The abrupt transition from rest to activity in muscle. *Proc. R. Soc. Lond. Ser. B Biol. Sci.* 136 399–420. 10.1098/rspb.1949.0033 18143369

[B36] Hillege M. M. Galli Caro R. A. Offringa C. de Wit G. M. Jaspers R. T. Hoogaars W. M. (2020). TGF-β Regulates Collagen Type I expression in Myoblasts and Myotubes via Transient Ctgf and Fgf-2 expression. *Cells* 9:375 10.3390/cells9020375 PMC7072622 32041253

[B37] Hornberger T. A. Esser K. A. (2004). Mechanotransduction and the regulation of protein synthesis in skeletal muscle. *Proc. Nutr. Soc.* 63 331–335. 10.1079/PNS2004357 15294051

[B38] Hornberger T. A. Chu W. K. Mak Y. W. Hsiung J. W. Huang S. A. Chien S. (2006). The role of phospholipase D and phosphatidic acid in the mechanical activation of mTOR signaling in skeletal muscle. *Proc. Natl. Acad. Sci. U.S.A.* 103 4741–4746. 10.1073/pnas.0600678103 16537399 PMC1450240

[B39] Huijing P. A. (2009). Epimuscular myofascial force transmission: a historical review and implications for new research. International Society of Biomechanics Muybridge Award Lecture, Taipei, 2007. *J. Biomech.* 42 9–21. 10.1016/j.jbiomech.2008.09.027 19041975

[B40] Huijing P. A. Jaspers R. T. (2005). Adaptation of muscle size and myofascial force transmission: a review and some new experimental results. *Scand. J. Med. Sci. Sports* 15 349–380. 10.1111/j.1600-0838.2005.00457.x 16293149

[B41] Hyldahl R. D. Nelson B. Xin L. Welling T. Groscost L. Hubal M. J. (2015). Extracellular matrix remodeling and its contribution to protective adaptation following lengthening contractions in human muscle. *FASEB J.* 29 2894–2904. 10.1096/fj.14-266668 25808538

[B42] Ito N. Ruegg U. T. Kudo A. Miyagoe-Suzuki Y. Takeda S. I. (2013). Activation of calcium signaling through Trpv1 by nNOS and peroxynitrite as a key trigger of skeletal muscle hypertrophy. *Nat. Med.* 19 101 10.1038/nm.3019 23202294

[B43] Jackson D. S. Bentley J. P. (1960). On the significance of the extractable collagens. *J. Cell Biol.* 7 37–42. 10.1083/jcb.7.1.37 PMC2224869 14406281

[B44] Järvinen T. A. Józsa L. Kannus P. Järvinen T. L. Järvinen M. (2002). Organization and distribution of intramuscular connective tissue in normal and immobilized skeletal muscles. *J. Muscle Res. Cell Motil.* 23 245–254. 10.1023/A:1020904518336 12500904

[B45] Jaspers R. T. van Beek-Harmsen B. J. Blankenstein M. A. Goldspink G. Huijing P. A. van der Laarse W. J. (2008). Hypertrophy of mature Xenopus muscle fibres in culture induced by synergy of albumin and insulin. *Pflügers Arch. Eur. J. Physiol.* 457:161 10.1007/s00424-008-0499-0 18493787

[B46] Jozsa L. Thöring J. Järvinen M. Kannus P. Lehto M. Kvist M. (1988). Quantitative alterations in intramuscular connective tissue following immobilization: an experimental study in the rat calf muscles. *Exp. Mol. Pathol.* 49 267–278. 10.1016/0014-4800(88)90039-1 3169207

[B47] Juffer P. Bakker A. D. Klein-Nulend J. Jaspers R. T. (2014). Mechanical loading by fluid shear stress of myotube glycocalyx stimulates growth factor expression and nitric oxide production. *Cell Biochem. Biophys.* 69 411–419. 10.1007/s12013-013-9812-4 24402674

[B48] Kim J. Lee J. (2017). Role of transforming growth factor-β in muscle damage and regeneration: focused on eccentric muscle contraction. *J. Exerc. Rehabil.* 13:621 10.12965/jer.1735072.536 PMC5747195 29326892

[B49] Kjaer M. (2004). Role of extracellular matrix in adaptation of tendon and skeletal muscle to mechanical loading. *Physiol. Rev.* 84 649–698. 10.1152/physrev.00031.2003 15044685

[B50] Kragstrup T. W. Kjaer M. Mackey A. L. (2011). Structural, biochemical, cellular, and functional changes in skeletal muscle extracellular matrix with aging. *Scand. J. Med. Sci. Sports* 21 749–757. 10.1111/j.1600-0838.2011.01377.x 22092924

[B51] Kristensen L. Therkildsen M. Riis B. Sørensen M. T. Oksbjerg N. Purslow P. P. (2002). Dietary-induced changes of muscle growth rate in pigs: effects on in vivo and postmortem muscle proteolysis and meat quality. *J. Anim. Sci.* 80 2862–2871. 10.2527/2002.80112862x 12462253

[B52] Krivickas L. S. Dorer D. J. Ochala J. Frontera W. R. (2011). Relationship between force and size in human single muscle fibres. *Exp. Physiol.* 96 539–547. 10.1113/expphysiol.2010.055269 21317219

[B53] Kühl U. Timpl R. von der Mark K. (1982). Synthesis of type IV collagen and laminin in cultures of skeletal muscle cells and their assembly on the surface of myotubes. *Dev. Biol.* 93 344–354. 10.1016/0012-1606(82)90122-1 7141102

[B54] Lacourpaille L. Hug F. Bouillard K. Hogrel J. Y. Nordez A. (2012). Supersonic shear imaging provides a reliable measurement of resting muscle shear elastic modulus. *Physiol. Meas.* 33:N19 10.1088/0967-3334/33/3/N19 22370174

[B55] Lännergren J. Westerblad H. (1987). The temperature dependence of isometric contractions of single, intact fibers dissected from a mouse foot muscle. *J. Physiol.* 390 285–293. 10.1113/jphysiol.1987.sp016700 3443937 PMC1192180

[B56] Lännergren J. Westerblad H. (1991). Force decline due to fatigue and intracellular acidification in isolated fibers of mouse skeletal muscle. *J. Physiol.* 434 307–322. 10.1113/jphysiol.1987.sp016700 1902515 PMC1181419

[B57] Latorre M. E. Palacio M. I. Velázquez D. E. Purslow P. P. (2019). Specific effects on strength and heat stability of intramuscular connective tissue during long time low temperature cooking. *Meat Sci.* 153 109–116. 10.1016/j.meatsci.2019.03.016 30925447

[B58] Lehmann K. B. (1907). Studies of the causes for the toughness of meats. *Arch. Hyg.* 3 134–142.

[B59] Lewis G. J. Purslow P. P. (1989). The strength and stiffness of perimysial connective tissue isolated from cooked beef muscle. *Meat Sci.* 26 255–269. 10.1016/0309-1740(89)90011-9 22055022

[B60] Lewis G. J. Purslow P. P. (1990). Connective tissue differences in the strength of cooked meat across the muscle fibre direction due to test specimen size. *Meat Sci.* 28 183–194. 10.1016/0309-1740(90)90002-N 22055572

[B61] Li Y. Huard J. (2002). Differentiation of muscle-derived cells into myofibroblasts in injured skeletal muscle. *Am. J. Pathol.* 161 895–907. 10.1016/S0002-9440(10)64250-2 12213718 PMC1867256

[B62] Light N. Champion A. E. (1984). Characterization of muscle epimysium, perimysium and endomysium collagens. *Biochem. J.* 219 1017–1026. 10.1042/bj2191017 6743238 PMC1153576

[B63] Light N. Champion A. E. Voyle C. Bailey A. J. (1985). The role of epimysial, perimysial and endomysial collagen in determining texture in six bovine muscles. *Meat Sci.* 13 137–149. 10.1016/0309-1740(85)90054-3 22055631

[B64] Lima K. M. M. Júnior J. F. S. C. de Albuquerque Pereira W. C. de Oliveira L. F. (2018). Assessment of the mechanical properties of the muscle-tendon unit by supersonic shear wave imaging elastography: a review. *Ultrasonography* 37 3–15. 10.14366/usg.17017 28607322 PMC5769952

[B65] Liu A. Nishimura T. Takahashi K. (1995). Structural weakening of intramuscular connective tissue during post mortem ageing of chicken semitendinosus muscle. *Meat Sci.* 39 135–142. 10.1016/0309-1740(95)80015-8 22059771

[B66] Loyd E. J. Hiner R. L. (1959). Meat Tenderness, Relation between Hydroxyproline of Alkali-Insoluble protein and tenderness of bovine muscle. *J. Agric. Food Chem.* 7 860–862. 10.1021/jf60106a010

[B67] Maas H. (2019). Significance of epimuscular myofascial force transmission under passive muscle conditions. *J. Appl. Physiol.* 126 1465–1473. 10.1152/japplphysiol.00631.2018 30605398

[B68] Maas H. Sandercock T. G. (2010). Force transmission between synergistic skeletal muscles through connective tissue linkages. *Biomed. Res. Int.* 2010:575672 10.1155/2010/575672 PMC2853902 20396618

[B69] Mackey A. L. Brandstetter S. Schjerling P. Bojsen-Moller J. Qvortrup K. Pedersen M. M. (2011). Sequenced response of extracellular matrix deadhesion and fibrotic regulators after muscle damage is involved in protection against future injury in human skeletal muscle. *FASEB J.* 25 1943–1959. 10.1096/fj.10-176487 21368102 PMC3101036

[B70] Mackey A. L. Magnan M. Chazaud B. Kjaer M. (2017). Human skeletal muscle fibroblasts stimulate in vitro myogenesis and in vivo muscle regeneration. *J. Physiol.* 595 5115–5127. 10.1113/JP274403 28369879 PMC5538230

[B71] Mackley J. R. Ando J. Herzyk P. Winder S. J. (2006). Phenotypic responses to mechanical stress in fibroblasts from tendon, cornea and skin. *Biochem. J.* 396 307–316. 10.1042/BJ20060057 16492137 PMC1462727

[B72] Magid A. Law D. J. (1985). Myofibrils bear most of the resting tension in frog skeletal muscle. *Science* 230 1280–1282. 10.1126/science.4071053 4071053

[B73] Mahdy M. A. A. (2019). Skeletal muscle fibrosis: an overview. *Cell Tissue Res.* 375 575–588. 10.1007/s00441-018-2955-2 30421315

[B74] Mannello F. Medda V. (2012). Nuclear localization of matrix metalloproteinases. *Progr. Histochem. Cytochem.* 47 27–58. 10.1016/j.proghi.2011.12.002 22226510

[B75] Manso A. M. Elsherif L. Kang S. M. Ross R. S. (2006). Integrins, membrane-type matrix metalloproteinases and ADAMs: potential implications for cardiac remodeling. *Cardiovasc. Res.* 69 574–584. 10.1016/j.cardiores.2005.09.004 16253214

[B76] Marcucci L. Bondı‘M. Randazzo G. Reggiani C. Natali A. N. Pavan P. G. (2019). Fibre and extracellular matrix contributions to passive forces in human skeletal muscles: an experimental based constitutive law for numerical modelling of the passive element in the classical Hill-type three element model. *PLoS One* 14:e0224232 10.1371/journal.pone.0224232 PMC6830811 31689322

[B77] Martineau L. C. Gardiner P. F. (2001). Insight into skeletal muscle mechanotransduction: MAPK activation is quantitatively related to tension. *J. Appl. Physiol.* 91 693–702. 10.1152/jappl.2001.91.2.693 11457783

[B78] Mathewson M. A. Chambers H. G. Girard P. J. Tenenhaus M. Schwartz A. K. Lieber R. L. (2014). Stiff muscle fibers in calf muscles of patients with cerebral palsy lead to high passive muscle stiffness. *J. Orthop. Res.* 32 1667–1674. 10.1002/jor.22719 25138654

[B79] McCormick R. J. (1989). “The influence of nutrition on collagen metabolism and stability,” in *Proceedings of the Reciprocal Meat Conference*, Laramie, Vol. 42 137–148.

[B80] McCormick R. J. (1994). The flexibility of the collagen compartment of muscle. *Meat Sci.* 36 79–91. 10.1016/0309-1740(94)90035-3 22061454

[B81] Meyer G. Lieber R. L. (2018). Frog muscle fibers bear a larger fraction of passive muscle tension than mouse fibers. *J. Exp. Biol.* 221:jeb–182089. 10.1242/jeb.182089 PMC6262763 30237238

[B82] Miller B. F. Wolff C. A. Peelor F. F. III Shipman P. D. Hamilton K. L. (2015). Modeling the contribution of individual proteins to mixed skeletal muscle protein synthetic rates over increasing periods of label incorporation. *J. Appl. Physiol.* 118 655–661. 10.1152/japplphysiol.00987.2014 25593288 PMC4360018

[B83] Mitchell H. H. Zimmerman R. L. Hamilton T. S. (1927). The determination of the amount of connective tissue in meat. *J. Anim. Sci.* 1927 257–257. 10.2527/jas1927.19271257x

[B84] Mutch S. (2015). “Myofascial force transmission,” in *Fascia in Sport and Movement*, Chap. 2, eds Schliep R. Baker A. Avison J. (Edinburgh: Handspring Publishing).

[B85] Nakamura M. Ikezoe T. Umegaki H. Kobayashi T. Nishisita S. Ichihashi N. (2016). Shear elastic modulus is a reproducible index reflecting the passive mechanical properties of medial gastrocnemius muscle belly. *Acta Radiol. Open* 5:2058460115604009 10.1177/2058460115604009 PMC4853831 27170845

[B86] Nishimura T. Hattori A. Takahashi K. (1994). Ultrastructure of the intramuscular connective tissue in bovine skeletal muscle. *Acta Anat.* 151 250–257. 10.1159/000147671 7740920

[B87] Olsen L. A. Nicoll J. X. Fry A. C. (2019). The skeletal muscle fiber: a mechanically sensitive cell. *Eur. J. Appl. Physiol.* 119 333–349. 10.1007/s00421-018-04061-x 30612167

[B88] Pamuk U. Karakuzu A. Ozturk C. Acar B. Yucesoy C. A. (2016). Combined magnetic resonance and diffusion tensor imaging analyses provide a powerful tool for in vivo assessment of deformation along human muscle fibers. *J. Mech. Behav. Biomed. Mater.* 63 207–219. 10.1016/j.jmbbm.2016.06.031 27429070

[B89] Passerieux E. Rossignol R. Chopard A. Carnino A. Marini J. F. Letellier T. (2006). Structural organization of the perimysium in bovine skeletal muscle: junctional plates and associated intracellular subdomains. *J. Struct. Biol.* 154 206–216. 10.1016/j.jsb.2006.01.002 16503167

[B90] Passerieux E. Rossignol R. Letellier T. Delage J. P. (2007). Physical continuity of the perimysium from myofibers to tendons: involvement in lateral force transmission in skeletal muscle. *J. Struct. Biol.* 159 19–28. 10.1016/j.jsb.2007.01.022 17433715

[B91] Purslow P. P. (1989). Strain-induced reorientation of an intramuscular connective tissue network: implications for passive muscle elasticity. *J. Biomech.* 22 21–31. 10.1016/0021-9290(89)90181-4 2914969

[B92] Purslow P. P. (1999). “The intramuscular connective tissue matrix and cell/matrix interactions in relation to meat toughness,” in *Proceedings 45th International Congress of Meat Science and Technology, 1-6 August 1999*, Vol. 1 Yokohama, 210–219.

[B93] Purslow P. P. (2002). The structure and functional significance of variations in the connective tissue within muscle. *Compar. Biochem. Physiol. Part A* 133 947–966. 10.1016/S1095-6433(02)00141-1 12485685

[B94] Purslow P. P. (2005). Intramuscular connective tissue and its role in meat quality. *Meat Sci.* 70 435–447. 10.1016/j.meatsci.2004.06.028 22063743

[B95] Purslow P. P. (2010). Muscle fascia and force transmission. *J. Bodywork Mov. Ther.* 14 411–417. 10.1016/j.jbmt.2010.01.005 20850050

[B96] Purslow P. P. (2014). New developments on the role of intramuscular connective tissue in meat toughness. *Ann. Rev. Food Sci. Technol.* 5 133–153. 10.1146/annurev-food-030212-182628 24437687

[B97] Purslow P. P. Archile-Contreras A. C. Cha M. C. (2012). Meat science and muscle biology symposium: manipulating meat tenderness by increasing the turnover of intramuscular connective tissue. *J. Anim. Sci.* 90 950–959. 10.2527/jas.2011-4448 21890505

[B98] Purslow P. P. Delage J. P. (2012). “General anatomy of the muscle fasciae,” in *Fascia: The Tensional Network of the Human Body*, eds Schleip R. Findley T. W. Chaitow L. Huijing P. A. (London: Churchill Livingstone), 5–10.

[B99] Purslow P. P. Duance V. C. (1990). “Structure and function of intramuscular connective tissue,” in *Connective Tissue Matrix*, ed. Hukins D. W. L. (London: Palgrave McMillan), 127–166.

[B100] Purslow P. P. Trotter J. A. (1994). The morphology and mechanical properties of endomysium in series-fibred muscles: variations with muscle length. *J. Muscle Res. Cell Motil.* 15 299–308. 10.1007/BF00123482 7929795

[B101] Ramsbottom J. M. Strandine E. J. Koonz C. H. (1945). Comparative tenderness of representative beef muscles. *Food Res.* 10 497–509. 10.1111/j.1365-2621.1945.tb16198.x 21007041

[B102] Roberts T. J. Eng C. M. Sleboda D. A. Holt N. C. Brainerd E. L. Stover K. K. (2019). The multi-scale, three-dimensional nature of skeletal muscle contraction. *Physiology* 34 402–408. 10.1152/physiol.00023.2019 31577172 PMC7002870

[B103] Rompala R. E. Jones S. D. (1984). Changes in the solubility of bovine intramuscular collagen due to nutritional regime. *Growth* 48 466–472. 6532904

[B104] Rowe R. W. D. (1974). Collagen fibre arrangement in intramuscular connective tissue. Changes associated with muscle shortening and their possible relevance to raw meat toughness measurements. *Int. J. Food Sci. Technol.* 9 501–508. 10.1111/j.1365-2621.1974.tb01799.x

[B105] Rowe R. W. D. (1981). Morphology of perimysial and endomysial connective tissue in skeletal muscle. *Tissue Cell* 13 681–690. 10.1016/S0040-8166(81)80005-5 7330851

[B106] Rowe R. W. D. (1986). Elastin in bovine Semitendinosus and Longissimus dorsi muscles. *Meat Sci.* 17 293–312. 10.1016/0309-1740(86)90047-1 22055360

[B107] Sakamoto Y. (1996). Histological features of endomysium, perimysium and epimysium in rat lateral pterygoid muscle. *J. Morphol.* 227 113–119. 10.1002/(SICI)1097-4687(199601)227 8568905

[B108] Schiaffino S. Dyar K. A. Ciciliot S. Blaauw B. Sandri M. (2013). Mechanisms regulating skeletal muscle growth and atrophy. *FEBS J.* 280 4294–4314. 10.1111/febs.12253 23517348

[B109] Schmalbruch H. (1974). The sarcolemma of skeletal muscle fibres as demonstrated by a replica technique. *Cell Tissue Res.* 150 377–387. 10.1007/BF0022014 4602399

[B110] Schmalbruch H. (1985). *Skeletal Muscle.* Berlin: Springer-Verlag, 5608–5609.

[B111] Scott I. Yamauchi M. Sricholpech M. (2012). Lysine post-translational modifications of collagen. *Essays Biochem.* 52 113–133. 10.1042/bse0520113 22708567 PMC3499978

[B112] Sharafi B. Blemker S. S. (2010). A micromechanical model of skeletal muscle to explore the effects of fiber and fascicle geometry. *J. Biomech.* 43 3207–3213. 10.1016/j.jbiomech.2010.07.020 20846654 PMC2993247

[B113] Sharafi B. Blemker S. S. (2011). A mathematical model of force transmission from intrafascicularly terminating muscle fibers. *J. Biomech.* 44 2031–2039. 10.1016/j.jbiomech.2011.04.038 21676398 PMC3134549

[B114] Shenkman B. S. Nemirovskaya T. L. Lomonosova Y. N. (2015). No-dependent signaling pathways in unloaded skeletal muscle. *Front. Physiol.* 6:298 10.3389/fphys.2015.00298 PMC4628111 26582991

[B115] Shimokomaki M. Elsden D. F. Bailey A. J. (1972). Meat tenderness: age related changes in bovine intramuscular collagen. *J. Food Sci.* 37 892–896. 10.1111/j.1365-2621.1972.tb03696.x

[B116] Stecco C. Gagey O. Macchi V. Porzionato A. De Caro R. Aldegheri R. (2007). Tendinous muscular insertions onto the deep fascia of the upper limb. First part: anatomical study. *Morphologie* 91 29–37. 10.1016/j.morpho.2007.05.001 17574470

[B117] Strandine E. J. Koonz C. H. Ramsbottom J. M. (1949). A study of variations in muscles of beef and chicken. *J. Anim. Sci.* 8 483–494. 10.2527/jas1949.84483x

[B118] Street S. F. (1983). Lateral transmission of tension in frog myofibers: a myofibrillar network and transverse cytoskeletal connections are possible transmitters. *J. Cell. Physiol.* 114 346–364. 10.1002/jcp.1041140314 6601109

[B119] Therkildsen M. Riis B. Karlsson A. Kristensen L. Ertbjerg P. Purslow P. P. (2002). Compensatory growth response in pigs, muscle protein turn-over and meat texture: effects of restriction/realimentation period. *Anim. Sci.* 75 367–377. 10.1017/S1357729800053145

[B120] Toursel T. Stevens L. Granzier H. Mounier Y. (2002). Passive tension of rat skeletal soleus muscle fibers: effects of unloading conditions. *J. Appl. Physiol.* 92 1465–1472. 10.1152/japplphysiol.00621.2001 11896011

[B121] Trotter J. A. (1993). Functional morphology of force transmission in skeletal muscle. *Cells Tissues Organs* 146 205–222. 10.1159/000147459 8317197

[B122] Trotter J. A. Purslow P. P. (1992). Functional morphology of the endomysium in series fibered muscles. *J. Morphol.* 212 109–122. 10.1002/jmor.1052120203 1608046

[B123] Turkoglu A. N. Yucesoy C. A. (2016). Simulation of effects of botulinum toxin on muscular mechanics in time course of treatment based on adverse extracellular matrix adaptations. *J. Biomech.* 49 1192–1198. 10.1016/j.jbiomech.2016.03.002 26994785

[B124] Turrina A. Martínez-González M. A. Stecco C. (2013). The muscular force transmission system: role of the intramuscular connective tissue. *J. Bodywork Mov. Ther.* 17 95–102. 10.1016/j.jbmt.2012.06.001 23294690

[B125] Westerblad H. Duty S. Allen D. G. (1993). Intracellular calcium concentration during low-frequency fatigue in isolated single fibers of mouse skeletal muscle. *J. Appl. Physiol.* 75 382–388. 10.1152/jappl.1993.75.1.382 8397180

[B126] Wilke J. Hespanhol L. Behrens M. (2019). Is It All About the Fascia? A Systematic Review and Meta-analysis of the Prevalence of Extramuscular Connective Tissue Lesions in Muscle Strain Injury. *Orthopaed. J. Sports Med.* 7:2325967119888500 10.1177/2325967119888500 PMC6931154 31903399

[B127] Wozniak A. C. Anderson J. E. (2009). The dynamics of the nitric oxide release-transient from stretched muscle cells. *Int. J. Biochem. Cell Biol.* 41 625–631. 10.1016/j.biocel.2008.07.005 18694846

[B128] Yucesoy C. A. Huijing P. A. (2012). Specifically tailored use of the finite element method to study muscular mechanics within the context of fascial integrity: the linked fiber-matrix mesh model. *Int. J. Multiscale Comput. Eng.* 10 155–170. 10.1615/IntJMultCompEng.2011002356

[B129] Yucesoy C. A. Koopman B. H. Huijing P. A. Grootenboer H. J. (2002). Three-dimensional finite element modeling of skeletal muscle using a two-domain approach: linked fiber-matrix mesh model. *J. Biomech.* 35 1253–1262. 10.1016/S0021-9290(02)00069-6 12163314

[B130] Zhang C. Gao Y. (2012). Finite element analysis of mechanics of lateral transmission of force in single muscle fiber. *J. Biomech.* 45 2001–2006. 10.1016/j.jbiomech.2012.04.026 22682257 PMC3843153

